# Case Report: Ultrasound Sciatic and Saphenous Nerve Blocks for Tibial Malunion Surgical Correction in a Pediatric African Leopard (*Panthera pardus*)

**DOI:** 10.3389/fvets.2020.538883

**Published:** 2020-11-27

**Authors:** Giuliano Ravasio, Federica Alessandra Brioschi, Vanessa Rabbogliatti, Daniela Gioeni, Federica Di Cesare, Federico Corletto, Maurizio Oltolina, Liliana Carnevale

**Affiliations:** ^1^Department of Veterinary Medicine, Università degli Studi di Milano, Milan, Italy; ^2^Department of Health, Animal Science and Food Safety, Università degli Studi di Milano, Milan, Italy; ^3^Dick White Referrals, Cambridgeshire, United Kingdom; ^4^Head of Veterinary Service, Zoological Park “Le Cornelle”, Valbrembo, Italy

**Keywords:** locoregional anesthesia, pediatric, sciatic nerve, ultrasound, saphenous nerve, patient state index, zoo animals

## Abstract

Little information is available regarding ultrasound-guided locoregional anesthesia in non-domestic species. Locoregional techniques have been shown to reduce intraoperative anesthetic requirements and provide postoperative pain relief. Decreasing dosage of general anesthetics allows more stable cardiopulmonary function during anesthesia and reduces the probability of side effects. An 11-week-old African leopard (*Panthera pardus*) was referred for treatment of a malunion angular limb deformity secondary to a tibial and fibular fracture. The animal was scheduled to undergo angular correction of the tibia via closing wedge osteotomy and fixation with a locking plate system. Following preanesthetic medication and induction of general anesthesia, a saphenous nerve block (ropivacaine 0.5%; 0.15 ml/kg) was performed under ultrasound guidance and a sciatic nerve block (ropivacaine 0.5%; 0.15 ml/kg) was performed using ultrasound and a peripheral nerve stimulator. Intraoperative anesthetic plane was considered light, yet no abrupt cardiocirculatory changes were seen, nor was rescue analgesia required. This case report suggests that sciatic and saphenous blockade could therefore be recommended as part of a multimodal plan of analgesia for orthopedic surgeries in pediatric exotic felids.

## Introduction

Regional anesthesia is well-described and widely used in veterinary medicine in order to control stress response under general anesthesia, improve perioperative analgesia, reduce opioids administration together with their related side effects, and promote earlier recovery from anesthesia (1, 2). On the other hand, potential serious complications such as direct trauma to neurovascular structures or ventricular arrhythmias (3, 4) are also reported. Regional blocks were originally performed, blindly, potentially limiting their success rate (5). Use of ultrasound (US) has increased the accuracy of target nerve location when performing nerve blocks (6). Improved understanding of sonographic anatomy should reduce both the failure rate and the likelihood of adverse events.

In humans, only a selection of blocks used in adults was commonly used in pediatric practice, until US guidance allowed increase in the number of blocks performed in infants and children (7). The use of US guidance and its incorporation into the practice of regional anesthesia have considerably improved routine pediatric perioperative care, demonstrating that regional anesthesia can be safely performed in children with minimal risk of neurological damage (8). In veterinary medicine, peripheral US-guided anesthesia in adult animals is considered safe (1, 2), but no clinical studies report its use in pediatric animals.

The combined sciatic and saphenous nerve block produces selective anesthesia of the pelvic limb distal to the stifle in dogs and is considered suitable to provide intraoperative and postoperative analgesia (9). Location of the sciatic nerve using electrostimulation has been reported (5) and the US identification of this nerve in dogs has been described (10, 11). Other studies have described the saphenous nerve block using US guidance (9, 12). To the authors' knowledge, only two veterinary studies on wild felids describe peripheral nerve blocks for the thoracic limb, respectively, blinded and with use of nerve stimulation (NS) (13, 14), and one report on US-guided femoral and sciatic nerve block in a Bennett's Wallaby (*Macropus rufogriseus rufogriseus*) (15).

This case report describes the successful blockade of sciatic and saphenous nerves with ropivacaine, as part of balanced anesthesia in a pediatric African leopard (*Panthera pardus*) undergoing surgery for tibial-fibular malunion angular limb deformity correction.

## Case Presentation

A 10-week-old female African leopard, weighing 3.2 kg, was referred for a 40-day history of lameness in the right pelvic limb, with no description of trauma. Clinical examination under mild sedation of intramuscular combination of dexmedetomidine 5 μg/kg and ketamine 1 mg/kg revealed swelling at the cranio-medial aspect of the right tibial. Medio-lateral and caudo-cranial radiography views of the right pelvic limb confirmed a malunion of tibial and fibular diaphyseal fractures resulting in a tibial *varus/procurvatum* deformity, probably due to nutritional secondary hyperparathyroidism. On the basis of physical examination, hematology and biochemistry, and echocardiography, the leopard was deemed healthy (American Society of Anesthesiologists status II). Closing wedge osteotomy and internal fixation with a 2.7 locking plate system (Synthes, Ltd.) were scheduled 10 days after admission. Meanwhile, confinement in a small and controlled area, oral gabapentin (10 mg/kg twice daily) for analgesia, and administration of specific diet to correct nutritional hyperparathyroidism were instituted.

On the day of surgery, food was withheld for 4 h and water was not restricted prior to general anesthesia. The leopard (weighing 3.8 kg) received an intramuscular combination of 5 μg/kg dexmedetomidine, 2 mg/kg of ketamine, and 0.2 mg/kg of methadone, using a squeeze cage for feral cats, to achieve sufficient sedation to allow intravenous (IV) cephalic catheter (20 G) placement.

General anesthesia was induced with IV propofol 1% (1.8 mg/kg) to enable tracheal intubation with a 5-mm cuffed endotracheal tube (ETT). The ETT was connected, via a heat and moisture exchanger/filter (HME), to a rebreathing circuit and mechanical ventilation was provided with a small animal ventilator (Merlin Small Animal Ventilator) using a volume-cycled mode with *f* r set to maintain P_ECO2_ between 35 and 42 mmHg with a tidal volume (VT) of 35 ml. Peak inspiratory pressure (PIP) was maintained below 10 cmH_2_O and isoflurane in 60% oxygen was administered to maintain anesthesia, guided by a neuronal function monitor (SEDLine™, Masimo, Irvine, CA), throughout surgery.

A multiparameter monitor (S5 Compact Anesthesia Monitor; Datex-Ohmeda, Florida, USA) was used throughout the anesthetic period to monitor lead II electrocardiogram, capnography, end-tidal isoflurane, pulse-oximetry, esophageal temperature, and non-invasive arterial blood pressure until cannulation of the left dorsal pedal artery with a 22-gauge catheter permitted invasive arterial blood pressure measurement. A Masimo Radical 7 monitor was used to measure perfusion index (Pi) and Patient State index (PSi). Body temperature was maintained between 36 and 37.5°C using a warm air blanket (Bair Hugger 505 Warming Unit, 3M, Germany). Physiological parameters recorded during general anesthesia are summarized in Table 1. Ringer lactate solution was administered IV at 3 ml/kg/h. Intraoperative rescue analgesia was planned as bolus administration of fentanyl (3 μg/kg IV) in response to an increase of HR or mean invasive blood pressure (mIBP) >20%.

**Table 1:** Intra-anesthetic parameters in an 11-week-old female African leopard (*Panthera pardus*).

	**HR**	***f*r**	**dIBP**	**mIBP**	**sIBP**	**PSi**	**Pi**	**SpO_**2**_**	**T°**
Mean	108.5	19.1	75.2	92.6	121.3	36.7	0.82	99.1	36.9
SD	7.1	1	9.2	10	13.3	3.4	0.2	0.7	0.5
Min	98	18	65	78	105	28	0.65	98	36
Max	120	20	91	109	145	42	1.1	100	37.6

*HR, Heart rate; fr, respiratory rate in mechanical ventilation; dIBP, diastolic invasive blood pressure in mmHg; mIBP, mean invasive blood pressure in mmHg; sIBP, systolic invasive blood pressure in mmHg; PSi, Patient State index; Pi, Perfusion index; SpO_2_, blood oxygen saturation; T°, oesophageal temperature in °C*.

Anesthetic depth was monitored using PSi (SEDLine™, Masimo monitor) and by evaluating clinical signs such as palpebral reflex, eye position, and ear twitch reflex. After aseptic preparation of the lateral and medial thigh area, sciatic, and saphenous nerve blocks were performed.

To provide perioperative analgesia, US-guided sciatic and saphenous nerve blocks were attempted as previously described (16). An US machine (Sonosite^®^ M-Turbo, Fujifilm, Netherlands) equipped with a 25 mm linear-array transducer (10–6 MHz) was used.

**Figure 1:**
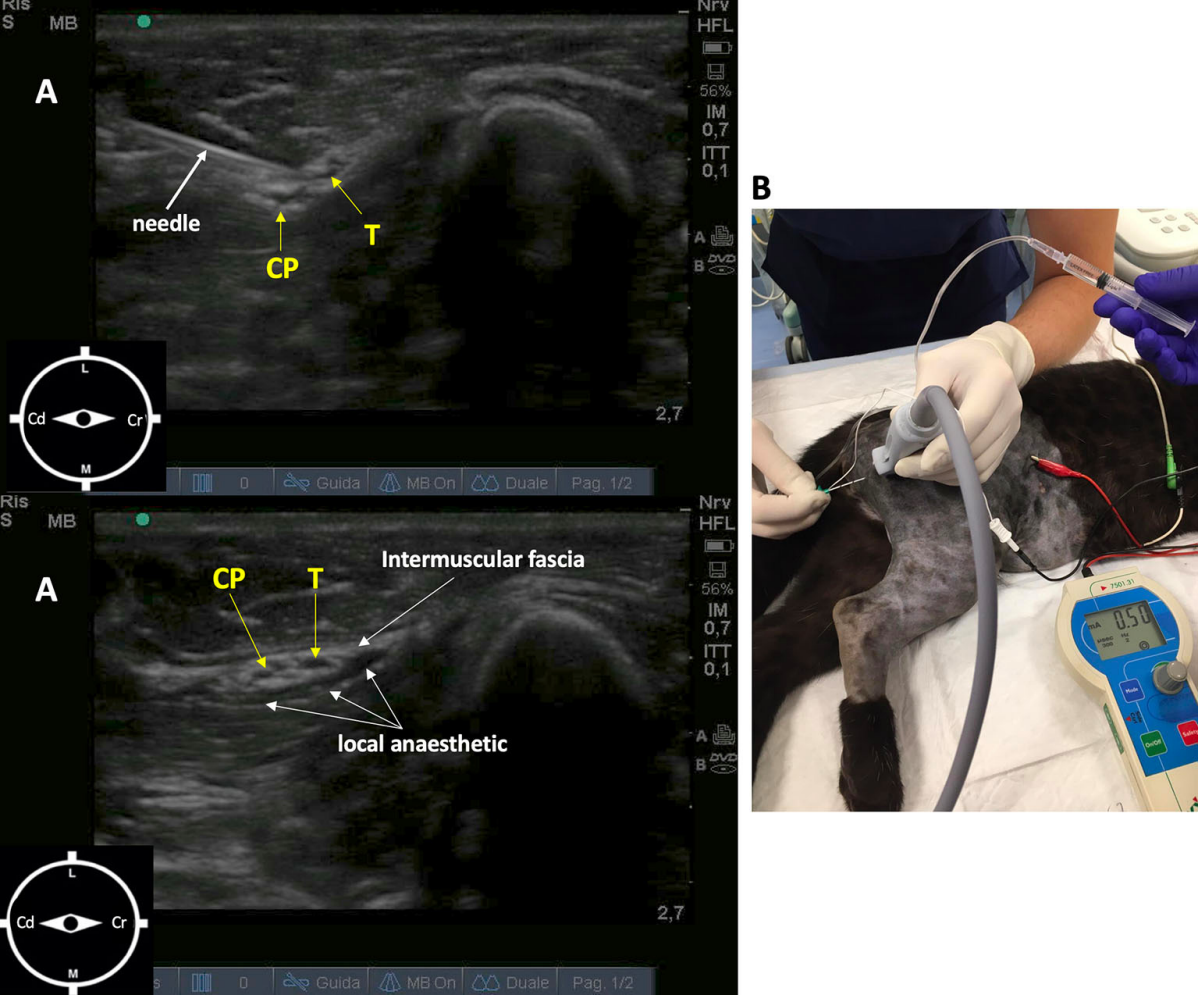
Ultrasound-guided sciatic nerve block, target muscular contraction to nerve stimulation in a 11-week-old female African leopard (*Panthera pardus*). **(A)** Acoustic window obtained with a linear transducer (10 MHz) positioned distal to the greater trochanter of the femur, with a transverse orientation to the long axis of the limb (L, lateral; M, medial; Cr, cranial; Cd, caudal). **(B)** Leopard positioned in lateral recumbency with the limb to be blocked uppermost. The needle is introduced in plane, and advanced in a cranial direction through the semitendinosus muscle, until its tip is positioned close to the perineural tissues. Nerve stimulation is used to achieve muscular response with >0.3 and <0.5 mA. CP, common peroneal nerve; T, tibial nerve.

The sciatic nerve block was performed with the leopard positioned in left lateral recumbency. The probe (10 MHz) was positioned distal to the femoral greater trochanter, with a transverse orientation to the long axis of the limb. The sciatic nerve showed two components, the tibial and common peroneal nerves, that appeared as round to oval, hypoechoic structures surrounded by hyperechoic connective tissue positioned cranially to greater trochanter. The needle (Bbrown^®^ 0.7 ×50 mm, 21-gauge ×30°), connected to a peripheral nerve stimulator (Plexygon Nerve Stimulator, Vygon^®^, France), was introduced in-plane and advanced in a cranial direction through the semitendinosus muscle, until its tip was positioned close to the sciatic perineural tissues and the threshold current for stimulation was >0.3 and <0.5 mA (16). Ropivacaine 0.5% (0.15 mg/kg) was administered after a negative aspiration test and in the absence of resistance to injection (Figure 1).

**Figure 2:**
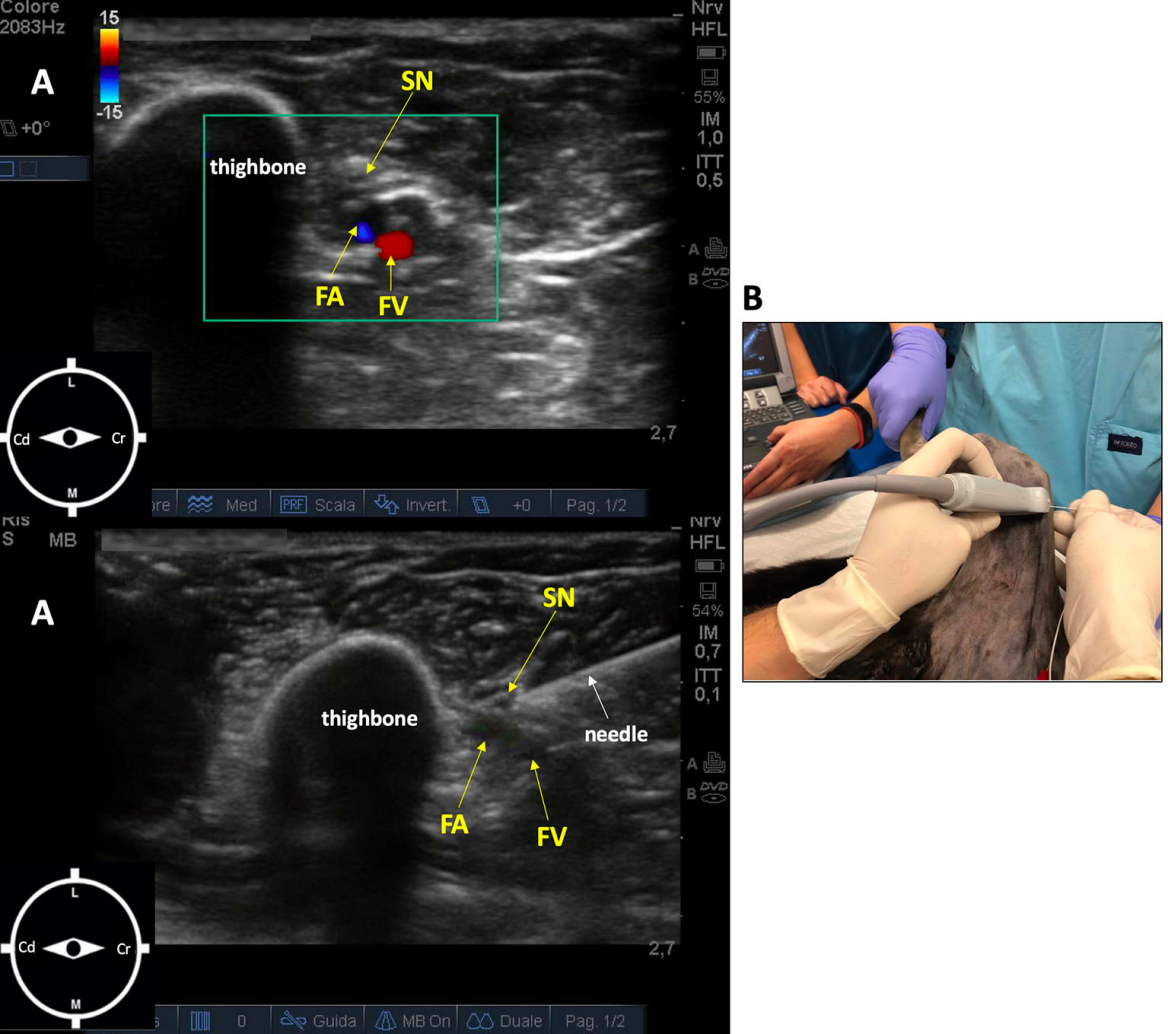
Ultrasound-guided saphenous nerve block in an 11-week-old female African leopard (*Panthera pardus*). **(A)** Acoustic window of the inguinal region obtained with a linear transducer (10 MHz), placed perpendicular to the long axis of the limb, at the level of the middle third of the thighbone. Note the femoral artery and vein, and the saphenous nerve cranial to them (L, lateral; M, medial; Cr, cranial; Cd, caudal). **(B)** Leopard positioned in lateral recumbency with the limb to be blocked uppermost, abducted 90°, and extended caudally. The needle is introduced in an in-plane approach and advanced in a craniocaudal direction. FA, femoral artery; FV, femoral vein; SN, saphenous nerve.

The saphenous nerve block was performed with the leopard in the same position, with the right pelvic limb abducted 90° and extended caudally. The probe (10 MHz) was placed perpendicular to the long axis of the limb, at the medial part of the limb at the level of the middle third of the femur. The femoral artery and vein were localized, and the saphenous nerve cranial to them. The needle (Bbrown^®^ 0.7 ×50 mm, 21-gauge ×30°) was introduced in an in-plane approach and advanced in a craniocaudal direction (16). Ropivacaine 0.5% (0.15 ml/kg) was administered after a negative aspiration test and in the absence of resistance to injection (Figure 2).

Physiological parameters remained stable intra-operatively (Table 1), and end-tidal isoflurane concentration ranged between 0.6 and 0.9%. The anesthetic plane was considered light during the surgical procedure, with persistent slight palpebral reflex, ventral rotation of the eye, and absence of swallowing reflex, muscle movement, lacrimation, and ear twitch reflex. However, rescue analgesic administration of fentanyl was not required. Total duration of anesthesia was 150 min, and surgery lasted 100 min. The ETT was removed 12 min after discontinuing isoflurane, when swallowing reflex appeared, and then the leopard was transferred to a dedicated cage to fully recover from anesthesia. Recovery was uneventful and no clear signs of discomfort or licking of the right pelvic limb were observed. Motor function of the quadriceps muscle appeared not to be affected, while paralysis and proprioception deficit distal to the stifle suggested motor and sensory blockade lasting for 6 h postoperatively. Postsurgical analgesia was achieved through a single injection of IM buprenorphine (15 μg/kg) and subcutaneous meloxicam (0.1 mg/kg), administered immediately after extubation. Gabapentin (10 mg/kg orally twice daily) was administered for 10 days, starting the day after surgery. Amoxicillin and clavulanic acid (20 mg/kg) were administered IV after placing the intravenous catheter and administered orally twice for 5 days after surgery. The postoperative physical and radiographic check at 6 weeks after surgery demonstrated correct position of the implant and bone healing, and the animal was no longer lame.

## Discussion

In recent years, use of US in human regional anesthesia has improved the success rate of peripheral nerve block and decreased the risk of potential complications including vascular puncture or nerve damage (17, 18). Limited information on the success rates and complications related to peripheral nerve block (1, 2) is published in companion animals, but encouraging results about US guidance in human regional anesthesia support its use in veterinary species.

The use of high-resolution US transducers allows the identification of small peripheral nerves as well as bony and vascular landmarks, the latter being distinguished using color-flow Doppler that provides an easy guide to avoid the blood vessels puncture during insertion of the needle. US-guided sciatic nerve block at the level of the proximal thigh has been described in both dogs and cats (11, 19). The sciatic and saphenous nerves were easily identified in this pediatric African leopard. In this animal, similarly to dogs, the sciatic nerve showed two components, the tibial and common peroneal nerves.

It has been reported (20) a motor deficit that lasted 18 h in 1 of 10 dogs after a sciatic nerve blockade with 0.3 ml/kg of a solution containing lidocaine 1% plus bupivacaine 0.25%, with complete resolution 30 h after treatment. In a retrospective evaluation of 265 sciatic nerve blocks in dogs, neurological complications were not identified at the 6-week postoperative examination (21). Self-mutilation of the dorsal aspect of the metatarsal region was reported in a dog that received a blind tibial and common peroneal nerve block (22); however, neurological damage during nerve block procedures is a very rare clinical condition (23). Actually, some exotic felids have been known to self-mutilate when in pain, so effective analgesia is essential (24).

The saphenous nerve is a small sensory nerve originating from the femoral nerve and can be blocked to provide anesthesia of the medial and cranial aspects of the stifle without affecting the motor function of the quadriceps muscle. Therefore, when this nerve is blocked in combination with a sciatic nerve block, the quadriceps muscle function can be preserved (2). This assumption is in agreement with clinical signs shown by the leopard after recovery: the motor function of the quadriceps muscle was preserved, while paralysis and proprioceptive deficit distal to the stifle were detected. These latter findings lasted for 6 h postoperatively, suggesting motor and sensory blockade.

Although US visualization of the saphenous nerve is not always possible in dogs (12), in this case, visualization of the nerve was judged satisfactory. In fact, the saphenous nerve was identified as a discontinuous, hyperechoic, oval structure, situated immediately cranial to the femoral artery (Figure 2). The US appearance of this nerve was more uniformly hyperechoic compared with the hypoechoic images observed in the sciatic nerve. The spread of ropivacaine was consistently seen using real-time US visualization as an anechoic space surrounding the nerves in both blocks.

The use of low volumes of local anesthetics avoids the potential of local and systemic toxicity, especially in small or pediatric animals when more than one block is performed (9). The volume of ropivacaine administered in the present study over the sciatic nerve (0.15 ml/kg) was in accordance with general literature recommendations (1, 2).

The combination between general anesthesia and peripheral nerve blocks is safer than general anesthesia alone (23). This strategy is directed to achieving the highest degree of efficacy while simultaneously limiting the amount and magnitude of undesired adverse effects that are associated with using any technique alone. Preemptive local anesthesia associated with general anesthesia improves patient comfort and should compare to systemic analgesia alone and should be routinely considered (23). In this case report, the intraoperative efficacy of the blocks was supported by the stability of physiological parameters; furthermore, no rescue drug was administered during surgery.

Although epidural anesthesia may be easier to perform than peripheral nerve blockade, the latter provides postoperative analgesia, which is comparable with those obtained with an epidural technique but with reduced side effects and is less likely to cause a severe neuraxial complication (25). Besides, unlike patients that receive epidural block, subjects that receive peripheral nerve blockade are improbable to experience urinary retention or discomfort about dragging pelvic limbs in the postoperative period (26).

In this case report, the anesthetic plan was constantly monitored by the anesthesiologist based on clinical signs. Additionally, the anesthetic depth was evaluated also with a PSi, a clinically validated measure of the effect of anesthesia and sedation in humans (27). This index extrapolated by EEG has been used in humans to reduce the amount of anesthetic drugs, decrease risk of awareness, and hasten recovery time (28, 29). The PSi values range from 0 (total cortical silence) to 100 (awake state), and 25–50 indicates the optimal hypnotic state for surgical anesthesia (30). This technology has not been validated in veterinary medicine and no studies in literature evaluate the correlation between PSi and specific anesthetic drugs. Unlike most anesthetics, ketamine increased Bispectral Index during anesthesia despite a deepening level of hypnosis (31). For the same reason, PSi should be interpreted carefully when this anesthetic agent is used as a part of a balanced protocol during anesthesia. The correlation between clinical signs and PSi values recorded in the leopard allowed us to hypothesize that this index could be useful in helping the veterinary anesthesiologist to evaluate the hypnosis depth of the animal. In fact, throughout the general anesthesia, the PSi value was on average 37 (range: 28–42) and the anesthetic plane was always stable without signs of awakening or deeper anesthesia. During recovery, just before swallowing reflex appearance, the PSi gradually increased up to 50, and at 70, it was possible to extubate the patient. After extubation, monitoring was stopped. Surgical maneuvers affecting the electrodes and muscle contractions may affect the quality of EEG signal and therefore the PSi reading. The monitor display signal quality to warn the user when the reading may be inaccurate. Future clinical trials using PSi should be performed in order to establish its actual role for anesthetic monitoring in veterinary patients.

Perfusion index was recorded via a Pulse CO-Oximeter^®^ (Masimo) positioned on the tongue. Perfusion index is an indirect and non-invasive measure of peripheral perfusion. This technology has been validated in human medicine, and its normal value ranged between 0.3 and 6 (32). In veterinary medicine, Pi has been found to be higher in dogs sedated with vasodilatory acepromazine compared to dexmedetomidine, which produces peripheral vasoconstriction (33). Moreover, an increase in Pi has been used to confirm successful blockade of the sciatic nerve by placing the probe on the lower extremity of the blocked pelvic limb in dogs (34). Throughout the intra-anesthetic period, this value remained between 0.65 and 1.1, assuming a suitable peripheral perfusion.

To avoid trauma or excitation during recovery, it was decided to introduce the animal in a small cage with a soft floor, in a low light and noiseless area. The leopard recovered spontaneously, was able to stand in about 30 min with no untoward effects, and showed appetite as early as 2 h after the end of surgery. In the postoperative 10-day period, no self-injuries or pain-related episodes were recorded, suggesting adequate postoperative analgesia.

## Concluding Remarks

In conclusion, sciatic and saphenous nerve block could be safely performed in this wild pediatric felid using the same technique used for domestic animals. These blocks, performed by using 0.15 ml/kg of ropivacaine 0.5% under US guidance, can be considered effective in providing intraoperative anti-nociception, characterized by excellent cardiovascular stability and halogenate sparing effect during surgery. Therefore, these peripheral nerve blocks can be suggested as part of a balanced anesthesia protocol for surgeries of the distal pelvic limb in pediatric wild felids.

## Data Availability Statement

The raw data supporting the conclusions of this article will be made available by the authors, without undue reservation.

## Ethics Statement

Ethical review and approval was not required for the animal study because the study was carried out in accordance with Italian and European laws. Written informed consent was obtained from the owner. The procedures performed are included as a part of the normal clinical management of patients.

## Author Contributions

GR designed research and wrote the first draft of the manuscript. GR, LC, DG, VR, FDC, and FAB contributed to methodology and validation. GR, LC, DG, VR, FDC, FC, MO, and FAB contributed to modify the manuscript and editing. All authors read and approved the final manuscript.

## Conflict of Interest

The authors declare that the research was conducted in the absence of any commercial or financial relationships that could be construed as a potential conflict of interest.
